# The Medicinal Prevention of Dental Decay

**Published:** 1899-04

**Authors:** George Howe Winkler


ARTICLE VII.

### THE MEDICINAL PREVENTION OF DENTAL DECAY.

BY GEORGE HOWE WINKLER, M. D., D. D. S.

Dental decay is a subject which, on first thought, would seem to be suitably assigned to the consideration and care of the dentist alone; but close study and successful treatment of this affection, extending through a period of nearly thirty years, convince me that the subject occupies a broader field and should be brought within the scope of the physician as well. The general practitioner is necessarily conversant with the diseases of humanity from the crown of the head to the soles of the feet, and is observant of the inter-dependence of pathological conditions in parts both adjacent and distant. This knowledge therefore renders his opinion in many cases of great value to a patient before the calling in of a specialist, and on this account I shall endeavor to show that dental caries come largely within the category of ills of which the medical adviser should have a broad comprehension.

The researches of Professor Miller, of Berlin, of Dr. J. Leon Wtlliams, of London, and Dr. G. V. Black, of Jacksonville, Ill., have demonstrated beyond any question that bacteria are present in all decay of the teeth, and their conclusion has been generally accepted that micro-organisms are the cause of dental decay. The slides exhibited recently in New York by Dr. Wiiliams have exposed these germs in the very act of their destructive work. Bacteria, wherever they can gain a lodgment in defects of the enamel or when in diseased condition of the mouth, can become felted or glued to the surface of the teeth, excrete lactic acid. This breaks down the calcium salts of the enamel and procures for the bacteria an opportunity to burrow into the enamel tissue or cement which binds the enamel rods together, and later on into the dentinal tubuli, excreting their acid as they burrow, thus producing decay. The conclusion has therefore been reached that these little organisms are the primary cause of dental caries. I do not accept this conclusion; on the contrary, I am convinced that while bacteria are present in all dental cavities, and are the direct cause of decay in some cases, the primary cause of dental decay in a majority of instances lies in a morbid state of the system which gives into the mouth perverted secretions. Close study of the subject, careful observation of the conditions of the mouth, and above all, successful prevention of decay by medicinal treatment, compel me to dissent from the generally accepted theory and enable me to divide the causes of dental caries into three classes. Two of these are pure dyscrasia, amenable to medicinal treatment. The third is a local disturbance requiring operative interference. The first class, which is the most destructive when it exists, consists of a pathological condition of the mucous glands of the mouth, more especially those situated on the gingival borders or free margins of the gums, which results in the excretions of an acrid corrosive fluid which excoriates the epithelium and corrodes the teeth, usually forming cavities around their necks. The mucous glands in a state of health secrete a clear, colorless liquid, very slightly acid, which bathes the necks and crowns of the teeth, dissolving and washing away any particles of food that may lodge about them. In the pathological condition which we are now considering this normal condition yields to the one above described, and apart from the severe damage done to the gums and teeth, not infrequently produces serious disturbance of digestion.

The appearance of the disease exhibits usually a line of inflammation extending along the festoons of the gums and interdental spaces, more frequently conspicuous about the palatal and lingual aspects of the molars, while at times the entire gingival borders are involved. The discoloration in some cases is so light as to require close scrutiny to detect it; at other times the diseased borders are turgid and purple in color; again, a crimson line marks the site of the affection, and in more advanced stages we have raw surfaces and phagedenic ulcerations.

The glandular excretion in some patients is clear, limpid, and sharply acid; the teeth appear clean, but present slight erosions at their necks, which are of the same color as the teeth, firm to the pressure of an instrument, but exquisitely sensitive; or the erosions may present the light-brown and sometimes the chalky-white appearance of rapid acid disintegration, soft in feeling, with pits here and there along the line of decalcification, and like the first, extremely sensitive. In other cases the excretion is turbid, viscid, excoriating, corrosive and fetid, covering the teeth with a murky or yellowish slime, which no amount of cleaning can keep off, imparting a disagreeable taste to the mouth, especially on waking in the morning, and an offensive odor to the breath. Beneath this tenacious exudate the teeth are covered with extensive decays, which present edges so friable as to be readily broken down by the pressure of the finger nail. So much for the first of my classes of the causes of dental caries; it is a simple dyscrasia and yields readily to medicinal treatment. Creosote in minute doses, frequently repeated, covering a period of from one to three weeks, is a specific for this pathological condition. The terrible destruction of dental tissue by corrosive excretions from the gingival borders is at once arrested by this remedy.

The cause of the second class of dental decay is also a morbid state of the system, which renders the fluids of the mouth destructive to the teeth and propitious to the enormous multiplication of micro-organisms, which, by their acid excretions and their borings, prove very destructive. The acid in the mouth in this class has rather more the characteristics of weak vegetable acids than the rapid corrosive action of mineral acids, such as the appearance of the first class always presents. The condition produces a vitiated state of the oral secretions, acid in reaction, and marked by an apparent lack of some deterrent element, wbich in normal saliva and mucous prevents the propagation and activity of bacteria. The latter, in the absence or lack of this deterrent, multiply enormously, and the acid condition of the mouth then may be attributable partly to the vitiated condition of the secretions and partly to the acid execretions of innumerable colonies of bacteria.

There are in the serum of the blood in health certain albuminous substances, pointed out by a number of eminent scientists, which have been called by Hankin

"defensive proteids." These proteids have been found in the serum of the blood of different animals, at times destructive to anthrax; again attenuating to the same germs—that is, shriveling and rendering them innocuous—and still again, in some cases, antitoxic or defensive against the toxic excretions of germs, upon which theory has been based the medication in the line of antitoxic treatment for various diseases which now occupies the most earnest attention of the secular as well as the medical mind.

Owing to the presence in healthy persons of these "defensive proteids," the secretions of the mucous surface are believed to be charged with a sufficient amount of some deterrent substance to render them safe against the attacks of micro-organisms which might be taken into the system. Every one living in a great city like New York receives more or less frequently into the alimentary, as well as the respiratory tracts, dust which is contaminated with various germs of disease; but these germs, coming into contact with the deterrent agent of normal mucous secretions furnished from the defensive proteids, are either destroyed or rendered innocuous and are cast out. If, however, a change of health occurs by which that function is held in abeyance which furnishes to our bodies the defensive proteids, and we are thus deprived of them, the germs which we take into us may inoculate us to our injury, or even to our death. The conditions which prevail in the mouth are as follews :

Bacteria are present in all mouths, healthy or diseased; they are present whether there is dental decay going on or whether there is not; at times they are in small numbers and apparently innocuous; again, they are found in numerous quantities, destroying the teeth with various degrees of rapidity, from a small number of cavities to an apparent melting away of the entire dental arch. They are in all mouths at all times, yet they are almost entirely in abeyance in some mouths, while they are rampant and destructive in others. In the same mouth we find them asserting no influence at some periods of life, while at other periods we find them quite destructive, I hold that these facts are due to the character of the secretions which bathe the mouth. When we are in a state of health, and our defensive proteids are in normal and protecting quantity, the bacteria are able to survive only in small numbers and in favorable locations; when a change occurs, and the peterrent element in the secretions is wanting, we suffer from the destructive agency of myriads of micro-organisms.

The appearance of the mouth in this class of ætiological factors shows generally very slight inflammation, although the gums are sometimes swollen and bleed very readily. The entire mouth is bathed in acid, except after some minutes of mastication, whereby the alkaline saliva has overcome the acid, and the teeth are affected by decay, more or less extensive, which is characterized by a very light-brown or chalky-white color and by rapid progress. The treatment of the above class is not so well defined as the first, and remedies have to be chosen with some discrimination from a number which are employed. These are very simple: The salts of mercury, potassium and calcium, charcoal, creosote, etc., are the most potent, administered in doses so small that there is no general systemic disturbance from their use; yet their action upon the diseased condition of the mouth is so pronounced as to be nearly specific.

Working on these lines of thought and with the above mentioned experience, I have been enabled to pursue a practice which has resulted in the prevention of dental decay to a most gratifying extent.

The third class in my division of the œtiology of dental caries constitutes that form of decay which is due not to a dyscrasia, but simply to acid-excreting bacteria which are lodged in sheltering localities about the teeth. The sulci in the crowns of molars and bicuspids, pits or defects in enamel, malformation of teeth, or a crowded condition of them, all afford lodgment for the bacteria of the mouth, and it is here only a question of time when their acid excretions will dissolve the lime salts and decay begin. The only treatment for this class is operative interference.

It seems to me, in conclusion, that the cause of decay of the teeth lies in abnormal or defective conditions. In the first two classes, in abnormal or defective conditions of the teeth themselves. To say that bacteria are the cause of dental decay sounds very much to me like saying that dyspepsia is the cause of dyspepsia. The earlier years of puberty and the period of gestation are especially the periods when the ravages of decay from the first two of the above classes are most pronounced, and then it is that the mouth should be most carefully watched and intelligent medicinal treatment be invoked to preserve the teeth. Treatment of from one to three weeks, according to severity, is sufficient to bring cases under control; in the case of young persons a re-examination should be made every three or four months during the first two or three years after the condition has manifested itself, and treatment be again resorted to if needed, for it is not certain that there may be a recurrence of the dyscrasis within this period.

Cleanliness of the teeth and mouth, and when the presence of acid prevails, the use of Philip's milk of magnesia, especially at night on retiring, is good. It covers the teeth with an alkaline film against which the acid for hours exhausts itself. This care is a repulse of the vitiated secretions after they have been poured out into the mouth. Such. medicinal treatment is at a stroke aimed at the fountain head (or source) of the infection, and when skillfully delivered frees the mouth at once of its foul condition and its acid excretions.

I am fully persuaded that more than fifty per cent. of dental caries is absolutely preventable by medicines internally administered, which act specifically in the mouth; alike pleasing and imperative is the appeal to both ambition and intelligence to prevent, rather than to repair, the ravages of decay.—*New York Medical Journal.*

